# Unusual Presentation of Esophageal Tuberculosis Mimicking Malignancy

**DOI:** 10.4103/1319-3767.39632

**Published:** 2008-04

**Authors:** Rashmi Patnayak, Mandyam K. Reddy, Sriram Parthasarathy, Mutheeswaraiah Yootla, Venkatarami Reddy, Amitabh Jena

**Affiliations:** Department of Pathology, Sri Venketeswar Institute of Medical Sciences, Tirupati - 517 507, Andhra Pradesh, India. E-mail: rashmipatnayak2002@yahoo.co.in; 1 Department of Surgical Gastroenterology, Sri Venketeswar Institute of Medical Sciences, Tirupati - 517 507, Andhra Pradesh, India; 2 Department of General Surgery, Sri Venketeswar Institute of Medical Sciences, Tirupati - 517 507, Andhra Pradesh, India

Sir,

Tuberculosis of the esophagus is rare in both immunocompetent and immunocompromised hosts. Esophageal tuberculosis usually occurs secondary to tuberculous infection of adjacent organs, such as lungs, larynx or mediastinum.[1] More cases of secondary tuberculosis of the esophagus have been reported compared with primary esophageal tuberculosis.[1] The diagnosis may be difficult as due to the lack of characteristic clinical features, imaging studies or laboratory findings. Even histology cannot always establish the diagnosis, usually leading to misdiagnosis as esophageal carcinoma.

A 75-year-old man presented with complaints of fever and vomiting of 1-week duration. Fever was intermittent, not nocturnal and not associated with chills and rigor. The patient did not suffer from any immunological disorders, was not on any immunosuppressive drug, and did not have any prior history of tuberculosis. He noticed a scalp swelling of 4-day duration, measuring 1 × 1 cm and soft in consistency. Upper gastrointestinal endoscopy revealed an excavating ulcer with undermined edges. The clinical suspicion was esophageal malignancy with probable scalp metastasis. Fine needle aspiration done from the scalp swelling yielded a pus-like material, the cytology smear of which showed acute and chronic inflammatory cells with epithelioid cell clusters and macrophages. Ziehl-Neelson stain demonstrated many acid-fast bacilli. Subsequently, the endoscopic biopsy specimen taken from the esophagus also revealed epithelioid granulomas.

Esophageal tuberculosis has various presentations. It generally affects the middle-third of the esophagus around the carina and is usually caused by direct extension and spread from mediastinal structures. Symptoms such as dysphagia and retrosternal pain are the most common complaints.[1–3] Diagnosing esophageal tuberculosis can be difficult and is usually discovered during surgery. Esophageal tuberculosis is suspected in patients with pulmonary or systemic tuberculosis who later develop dysphagia. Delay in diagnosis and instituting appropriate therapy might induce severe complications.[3,4]

Most cases can be successfully treated with antituberculous chemotherapy, even in the presence of fistulous tracts. The patient responded to treatment with antituberculous drugs.

In conclusion, it is difficult to distinguish esophageal tuberculosis from malignancy on clinical findings alone. We report an unusual presentation of disseminated esophageal tuberculosis mimicking metastatic malignancy. Fine-needle aspiration cytology demonstrating acid fast bacilli, along with histological demonstration of epitheloid granulomas on endoscopic specimens played an important role in diagnosing this case.

## References

[1] Jain SK, Jain S (2002). Esophageal tuberculosis: Is it so rare? Report of 12 cases and review of the literature. Am J Gastroenterol.

[2] Laajam M (1984). Primary tuberculosis of the esophagus: Pseudo-tumor presentation. Am J Gastroenterol.

[3] Robbs JV, Bhoola KD (1976). Aorto-oesophageal fistula complicating tuberculous aortitis. S Afr Med J.

[4] Iwamoto I, Tomita Y (1995). Esophagoaortic fistula caused by esophageal tuberculosis: Report of a case. Surg Today (Jpn J Surg).

